# A novel computational approach for study of proton–proton scattering

**DOI:** 10.1038/s41598-025-97309-4

**Published:** 2025-09-30

**Authors:** Arushi Sharma, Ayushi Awasthi, O. S. K. S. Sastri

**Affiliations:** Department of Physics and Astronomical Sciences, Central University of Himachal Pradesh, Dharamsala, India

**Keywords:** Physics, Nuclear physics, Theoretical nuclear physics

## Abstract

In this paper, we have implemented a novel computational approach to study proton-proton scattering. The approach is applicable to all charged particle scattering scenarios and solves the challenge of incorporating the long-range Coulomb interaction alongside the short nuclear interaction using the phase function method. The key idea is to construct a reference potential using three smoothly joined Morse functions that collectively capture both nuclear and electromagnetic interactions. The reference potential is utilized in solving the phase equation which is derived by the transformation of the Schrodinger equation, for obtaining the scattering phase shifts for different values of orbital angular momentum called as $\ell$-channels (S, P, D, F, G, H). The parameters of reference potential are optimized to minimize the Mean Squared Error between obtained and expected phase shifts, resulting in the construction of inverse potential for various $\ell$-channels of the proton-proton system. Utilizing the phase shifts obtained from the inverse potentials, we have calculated the total cross-section and the low energy effective-range parameters, which have been found to be in excellent match with the experimental data.

## Introduction

The theoretical way to explain the experimental scattering phenomenon are based on both direct and inverse methods. The direct methods involve modeling the underlying interactions such as central, spin, iso-spin, and orbital angular momentum and their mixing, with appropriate mathematical functions. The time-independent Schrodinger equation is solved for the model potential to obtain the corresponding wave functions from which one obtains the scattering phase shifts using various methods such as R-matrix^1^ and Jost functions^2^. Finally, the model parameters are adjusted such that the obtained scattering phase shifts match the experimentally obtained ones from scattering cross sections in the least-squares sense.

Alternatively, inverse scattering involves determining the scattering potential from the S-matrix elements associated with each orbital angular momentum $\ell$ and spin-orbit interactions. This approach can also extract the tensor terms relevant to coupled channel scenarios. The inverse methods for deriving the potential from the S-matrix are based on either fixed $\ell$ or fixed *E*(energy). The latter has many variations, such as Newton-Sabatier, WKB, and iterative perturbation^3^. Another approach to finding the inverse potential is to calculate the radial wave function for a selected partial wave $\ell$ that produces the correct S-matrix describing the observed scattering behavior. In this paper, our approach falls under the category of fixed $\ell$. The most significant inverse problems solved with fixed $\ell$ are by Gel’fand et al.^4–6^. These involve obtaining $S_\ell$(S-matrix element) for the whole energy range corresponding to different $\ell$. However, typically the available range of scattering energies is limited. In principle, all these methods are constrained by errors and may not always produce unique solutions. Thus, practically inverse methods involve solving either an under-determined or an over-determined set of equations on substituting scattering phase shift data which is obtained from scattering experiments.

The inverse technique being employed in this paper is based on the Phase Function Method^7–10^. This involves solving a set of first-order non-linear differential phase equations for various experimental scattering energies for each of the single and coupled channels. The chosen model potential is taken as input to directly obtain the scattering phase shifts without determining the wavefunctions.

When systems involve charged particles, the Coulomb interaction becomes significant because it extends over an infinite range compared to short-range forces like nuclear interactions. Solving this kind of long-range interaction using the phase function method requires consideration of Coulomb wavefunctions to be matched at the boundary of the potential. In an experimental scenario, isolated charges are typically surrounded by residual particles due to polarization. Thus, it is acceptable to model the electromagnetic repulsion using screened Coulomb potential, which has a power function such as $\alpha ^\rho$.

Taylor^11^ presented a comprehensive method of incorporating Coulomb scattering by considering

1 $$\begin{aligned} V_c^\rho (r) = \frac{\gamma }{r}\alpha ^\rho (r) \end{aligned}$$

where $\gamma$ is constant and the screening function $\alpha ^\rho (r)$ must go to zero as r $\rightarrow$ $\infty$ and must approach 1 as the screening radius $\rho \rightarrow \infty$ with r fixed. As long as $\rho$ is very large, such a potential meets the conditions of scattering theory and produces findings that are independent of both properties of screened potential, their nature/shape, and screening radius.

Generally, the screening for Coulomb interaction has been modeled using either an atomic Hulthen^12,13^ or an *erf()*^14–16^ function. The *erf()* function does not smoothly approach zero as r increases; instead, it necessitates an abrupt cutoff at a specific distance. Similarly, the atomic Hulthén potential requires adjusting the screening radius for different angular momentum $\ell$ channels, presenting a major limitation of these methods. In this paper, we propose a novel computational approach to overcome these challenges and accurately incorporate the Coulomb interaction without any additional Ansatz.

We have employed both these functions for studying various charged particle systems, such as proton-proton^9^, $\alpha -\alpha$^12,17^, proton-deuteron^18^, etc. Even though one obtains reasonably good results with these functions, they do not strictly satisfy the requirements proposed by Taylor^11^.

In literature, while modeling the underlying interaction, various physically relevant terms (such as central, spin-orbit, tensor, polarization term, etc.) are used which drives the success of direct theoretical methods. In contrast, the methodology of constructing inverse potentials does not need to adhere to the same considerations. The inverse methods are driven by the philosophy that the ‘whole’ need not necessarily be just a simple combination of the ’parts’. Hence, one need not restrict the choice of repulsive interaction being represented by the Coulomb function while implementing the inverse potential approach. So, the question arises: What could be an appropriate mathematical form that could correctly incorporate the repulsive nature that becomes important in charged particle scattering? Selg^19^ tackles this beautifully by constructing a piece-wise smooth combination of Morse functions for describing a interaction potential. The solution lies in choosing an inverse Morse function form for capturing the electromagnetic interaction that occurs in these systems. The number of Morse functions to be stitched together depends on the nature of the scattering system.

For instance, we have recently studied $\alpha -\alpha$ scattering using a combination of two piece-wise smooth Morse functions^20^ and have successfully obtained the inverse potentials for its various $\ell$ channels. Similarly, for the neutron-proton system, we have utilized a three-component Morse function as a reference to construct a high-precision inverse potential, and hence obtain accurate low-energy scattering parameters^21^. In this paper, we employ a similar approach to derive accurate total cross-sections and scattering parameters using the constructed inverse potential for proton-proton scattering.

Typically, the nucleon-nucleon potentials have been designed by various research groups such as Reid^22^, $Av_{18}$^23^, Nijm, and CD-Bonn^24^ by dividing the interaction into three regions as:

- long-range, $r\ge 2 fm$, modeled using one pion exchange potential (OPEP), including magnetic moment, vacuum polarization, and relativistic effects,
- intermediate-range, $0.5 \le r \le 2 fm$, modeled using scalar meson exchanges, which mediate attractive nuclear forces. These mesons effectively account for the binding effects between nucleons in this range.
- short-range, $r < 0.5 fm$, modeled using vector Boson exchanges along with QCD(Quantum Chromodynamics) effects, which account for the strong force between quarks and gluons.

All these phenomenological potentials consider central, spin-orbit, tensor, and a combination of spin, iso-spin, and orbital angular momentum components. In addition to these, the Argonne group^23^ has introduced, for the proton-proton system, alongside the electromagnetic interaction, various other terms such as one and two-photon Coulomb terms: Darwin-Foldy term, vacuum polarization, and magnetic moment interaction. Each of these terms has been multiplied by the appropriate form factor. They have also added a quadratic spin-orbit term to the nuclear interaction. So, every research group has added different types of mathematical terms based on certain physical considerations, resulting in a wide variety of approaches^3^. Yet, one can not be sure as to whether these interactions would finally be enough to represent the whole. Hence, the inverse potential approach has a better chance of succeeding in obtaining the underlying overall interaction in nucleon-nucleon scattering.

We have recently constructed inverse potentials for all the single and multi-channel scattering states of the neutron-proton system by utilizing a piece-wise smooth reference potential consisting of three Morse functions^21^. Similarly, we have constructed the piece-wise smooth reference potential for the charged particle system by choosing a proton-proton system.

The main objectives of our work are as follows:

- *Construction of a reference potential* Since proton-proton scattering has a repulsive term in addition to the nuclear interaction, the reference potential needs to be modified to include the possibility of the Coulomb barrier appearing at large distances. Therefore, we have chosen to construct the reference potential to consist of two Morse functions for the first two parts, representing the short and medium range, and an inverse Morse function, having a negative sign, for capturing the long-range interaction. The overall potential has the following form:
  2 $$\begin{aligned} U_{i}(r)= {\left\{ \begin{array}{ll} V_0 + D_0[e^{-2\alpha _0(r-r_0)}-2e^{-\alpha _0(r-r_0)}],& \text {if } r\le X_1\\ V_1 + D_1[e^{-2\alpha _1(r-r_1)}-2e^{-\alpha _1(r-r_1)}],& \text {if } X_1<r< X_2\\ V_2 - D_2[e^{-2\alpha _2(r-r_2)}-2e^{-\alpha _2(r-r_2)}],& \text {if}~ r\ge X_2 \end{array}\right. } \end{aligned}$$
  where *i* = 0, 1 and 2. Here $D_0, D_1 ~ \& ~D_2$ are potential depths at equilibrium distances $r_0, r_1~ \& ~ r_2$, and $\alpha _0, \alpha _1~ \& ~ \alpha _2$ reflect shape parameters of respective Morse functions. To obtain a smoothly varying potential, it must satisfy the boundary conditions at $X_1$ and $X_2$ to maintain continuity and smoothness criteria^20,21^. $U_0$, $U_1$ and $U_2$ have 4 parameters each. Further, $X_1$ and $X_2$ also need to be varied. So, it is a fairly complex potential with 14 parameters. Applying the four boundary conditions, one can obtain 4 parameters in terms of other parameters. Hence, the total number of the model parameters is reduced to 10. So, the reference potential is a family of curves with a total of 10 parameters including the boundary points $X_1$ and $X_2$, provide further flexibility and hence greater variety. Thus, we need to optimize 10 parameters to construct inverse scattering potentials for single channel scattering by solving the phase equation numerically, for various energies, in an iterative loop till the mean square error (MSE) between computed and experimental scattering phase shifts converges to a minimum value.
- *Solving the phase equations* This reference potential can be utilized as an input to the phase equation to obtain the phase shifts for the single-channel scattering for different $\ell$-values, given by
  3 $$\begin{aligned} \frac{d\delta _\ell (k,r)}{dr}=-\frac{U(r)}{k}\bigg [\cos (\delta _\ell (k,r)\hat{j}_{\ell }(kr)-\sin (\delta _\ell (k,r)\hat{\eta }_{\ell }(kr)\bigg ]^2 \end{aligned}$$
  where $U(r)=\frac{2\mu V(r)}{\hbar ^2}$. Here, $\hat{j}_{\ell }$ and $\hat{\eta }_{\ell }$ are the Riccati-Bessel functions and Riccati-Neumann functions^25^. The initial condition for the phase equation is $\delta _{\ell }(r = 0) = 0$. The final phase shift measured, $\delta _{\ell }(r \rightarrow \infty )$, represents the accumulated phase shift as the distance approaches infinity. Equation 3 is a non-linear equation comprises of Riccati-bessel and Riccati-Neumann functions which can be solved numerically using Runge-Kutta 5th order (RK-5) method.
- For the multi-channel scattering, we have solved three coupled non-linear differential equations using the Stapp parametrization^26^. These equations include the tensor forces for the spin-triplet states corresponding to different J-states of $\ell$ channel. For many channel scattering, we need to construct three potentials by solving three coupled non-linear first order differential equations simultaneously. For this, we need to optimize 30 parameters to obtain potentials corresponding to different total angular momenta, J, along with the tensor potential. For pp-scattering we have 1 S-state ($^1S_0$), 3 P-states ($^3P_0, ^3P_1~ \& ~ ^3P_2$), 1 D-state ($^1D_2$), 3 F-states ($^3F_2, ^3F_3~ \& ~ ^3F_4$), 1 G-state ($^1G_4$) and 1 H-state ($^3H_1$), a total of 10 states. Out of these, four states have mixing due to the inclusion of tensor potential, which results in two multi-channel states ($^3P_2, ^3F_2$) and ($^3F_4, ^3H_4$).
- *The low energy scattering parameters* Using the phase shifts obtained from the inverse potential, low-energy scattering parameters are determined by incorporating the electromagnetic interaction into the low-energy effective range expansion^27^, resulting in:
  4 $$\begin{aligned} C^2~ k~ \cot \delta _0 + (1/R)h(\eta )=-1/a_p + \frac{1}{2}r_e k^2 -Pr_e^3k^4 +Qr_e^5k^6 \ldots \end{aligned}$$
  where
  5 $$\begin{aligned} C^2=\frac{2\pi \eta -1}{e^{2\pi \eta }-1} \end{aligned}$$
  is the probability of finding two interacting charge particles at zero separation
  6 $$\begin{aligned} R=\frac{\hbar ^2}{M_pe^2} \end{aligned}$$
  and
  7 $$\begin{aligned} h(\eta )= \textrm{Re}\frac{\Gamma '(-i\eta )}{\Gamma (-i\eta )}-\textrm{ln}(\eta ) \end{aligned}$$
  k is the relative momentum in units of $\hbar$, $\eta =e^2/\hbar v$ is the Coulomb parameter, e is the proton charge, *v* is the relative velocity, and $a_p$ is the proton-proton scattering length, and $r_e$ is the effective range. *P* and *Q* are known as shape-dependent parameters, i.e. their values and sign depend on the detailed shape of the potential well in a Hamiltonian formulation or on model characteristics^28^.
- *Scattering cross sections* Once the Scattering Phase Shifts (SPS) $\delta _\ell (E)$ is obtained, for each orbital angular momentum $\ell$, one can calculate the partial Scattering Cross Section (SCS) $\sigma _\ell (E)$ using the formula^29^:
  8 $$\begin{aligned} \sigma _l(E;S,J)=\frac{4\pi }{k^2}\sum _{S=0}^{1}\left( \sum _{J=|\ell -S|}^{|\ell +S|}(2\ell +1)~\sin ^2(\delta _\ell (E;S,J)\right) \end{aligned}$$
  Additionally, the total Scattering Cross Section (SCS) $\sigma _T$ is given by:
  9 $$\begin{aligned} \sigma _T(E;S,J)=\frac{1}{\sum _{J=|\ell -S|}^{|\ell +S|}(2J+1)}\sum _{\ell =0}^{n}\sum _{S=0}^{1}(2J+1)\sigma _\ell (E;S,J) \end{aligned}$$
  Here, ’n’ represents the number of $\ell$-channels available for the scattering system. These equations allow for the calculation of partial and total scattering cross-sections based on the obtained scattering phase shifts.

## Results

### Choice of database

A comprehensive repository has been established for nucleon-nucleon scattering, encompassing measurements from research facilities worldwide. These datasets, originating from diverse laboratories, exhibit distinct statistical and systematic uncertainties that necessitate consideration when amalgamating them into a unified analysis. Among the most renowned repositories are the ENDF/B^30^, JENDL^31^, and George Washington (GW) Data Analysis Center^32^ nuclear data files. In the ENDF/B evaluation of Nucleon-Nucleon cross sections, an R-matrix analysis of the nucleon-nucleon system^1^ was employed. Conversely, in the JENDL Nucleon-Nucleon total cross-section assessment, a methodology founded on phase-shift data^33^ was adopted.

In this paper, we utilized the Granada database, which contains information up to the year 2013^34^. They have employed a refined criterion to ensure the selection of a coherent database consisting of $N = 6713$ sets of neutron-proton and proton-proton scattering data. The Granada database, known for its self-consistency, stands as the most extensive dataset to date that aligns with a statistically effective partial wave analysis of neutron–proton (np) and proton–proton (pp) scattering for laboratory energies up to 350 *MeV*. They have carefully considered all statistical versus systematic errors and have refined the database to comprise only 11 data points at energies (1, 5, 10, 25, 50, 100, 150, 200, 250, 300, and 350) *MeV* for each of these states and the mixing parameters for the multi-channel scattering states.

### Choice of sample space

The parameters of the reference potential in Eq. (2) are assigned certain bounds, thus defining the sample space for the family of curves. If the bounds are too broad, the sample space expands significantly, leading to longer computational times for convergence. Conversely, there is a possibility that the solution lies outside the chosen sample space if the bounds are too small. Sometimes, even if the solution converges with a reasonably small mean squared error(MSE) between the obtained and expected scattering phase shifts, the resulting solution might not accurately reflect the system’s physical characteristics.

### Construction of inverse potential

Once the parameter space is chosen, one picks a random set of initial parameters and constructs the reference potential from them. The phase equation or coupled equations for the respective single-channel or multi-channel scattering states are solved using the RK-5(Runge-Kutta) method. Using the obtained scattering phase shifts, the mean squared error is determined. The parameters are updated using a genetic algorithmic approach^20,21^. Then, the process is iterated till the mean squared error converges to a minimum value.

### Simulation of the results

#### Single-channel scattering

The benefit of employing a piece-wise smooth combination of Morse functions as a reference accrues from its three shape parameters($\alpha _0,~ \alpha _1~ \& ~ \alpha _2$), which allows for a large variety of curves, that result in the correct long-range aspect of the Coulomb interaction, while still preserving the deep attractive characteristics anticipated for the intermediate range due to nuclear force. To obtain the inverse potentials for single channels of proton-proton (pp) scattering using the reference potential approach, we need to optimize 10 model parameters of the potential, given in Eq. (2).

To begin with, the initial bounds are selected similarly to those used in neutron–proton interaction^21^ for the six single-channel states $^1S_0, ^3P_0, ^3P_1, ^1D_2, ^3F_3$, and $^1G_4$. The simulation has converged to result in inverse potentials with mean squared errors of 0.0017, 0.0002, 0.0008, $10^{-4}$, 0.0008, and 0.0003 respectively.

**Fig. 1:**
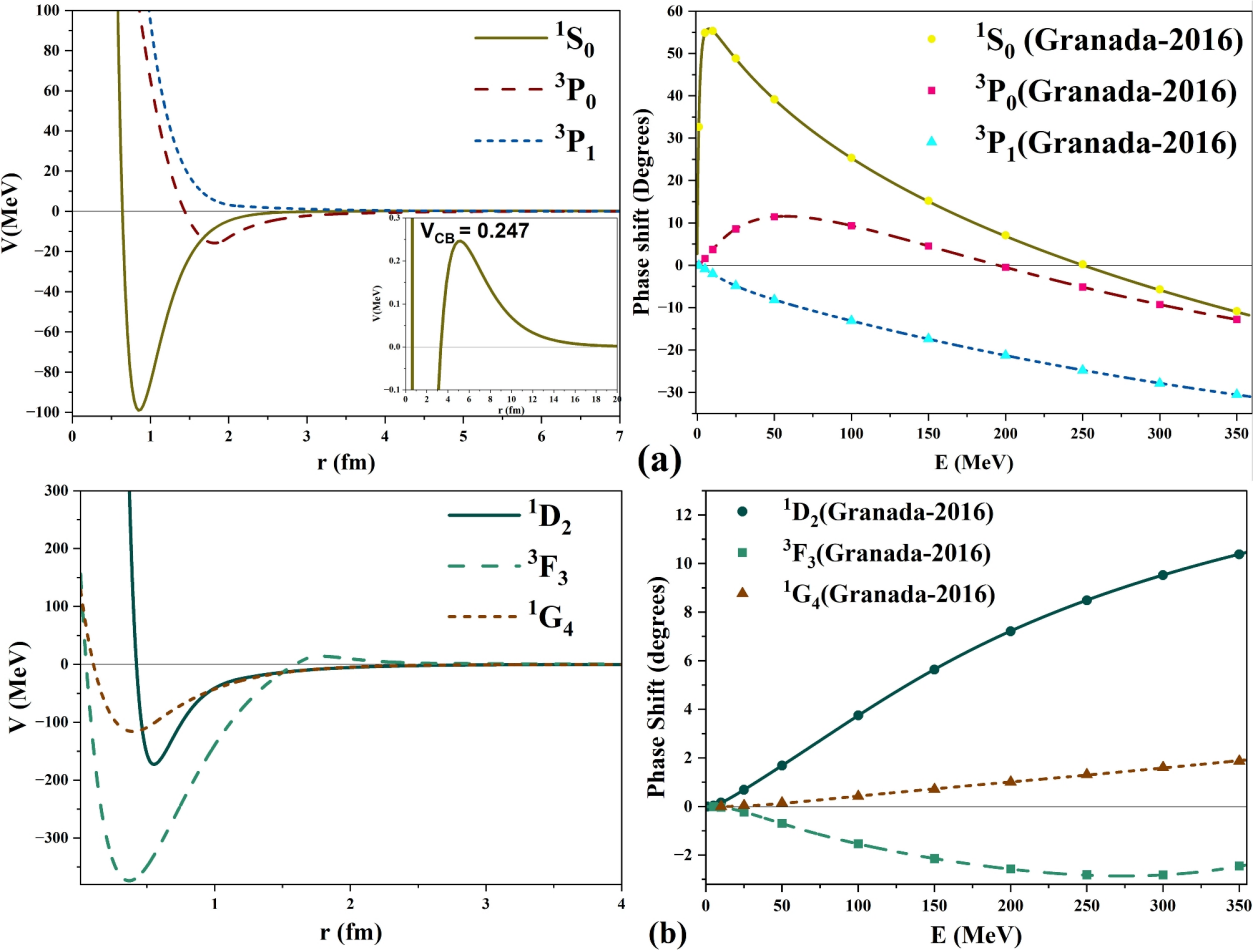
Inverse potentials and scattering phase shifts for the single channel scattering for (**a**) S and P waves, (**b**) D, F, and G waves.

The obtained inverse potentials for *S* and *P* waves and their corresponding scattering phase shifts are plotted in Fig. 1a. Similarly, Fig. 1b displays plots for the states *D*, *F*, and *G*.

It is observed that the depth of $^1S_0$ is close to 100 *MeV* and $^3P_0$ is about 20 *MeV*. On the other hand, the depths of $^1D_2$, $^3F_3$, and $^1G_4$ are more than 100, 150, and 350 *MeV* respectively. Typically, the depth of a potential reflects the magnitude and trend of phase shift. Positive scattering phase shifts will always lead to negative/attractive potentials. A larger positive phase shift indicates a deeper potential, while a smaller one corresponds to a shallower depth. Negative scattering phase shifts typically result in repulsive/positive potential. A decreasing trend in positive phase shifts indicates the presence of a repulsive core, whereas an increasing trend in negative phase shifts exhibits an attractive nature in the potential.

Considering that neutron–proton and proton–proton are mirror systems, one would anticipate their energy level structures and thus their inherent potentials to be identical. This can also be observed from the phase shift data of various j-states in both the neutron–proton and proton–proton systems, which are fairly similar at all scattering energies. We have observed that the obtained inverse potential for the proton-proton $^1S_0$ state has a similar shape and depth as that of the neutron-proton system. But, the inverse potentials obtained for $^1D_2, ^3F_3,$ and $^1G_4$ states of proton-proton are not similar to those obtained for the corresponding states in neutron–proton system^21^.

**Fig. 2:**
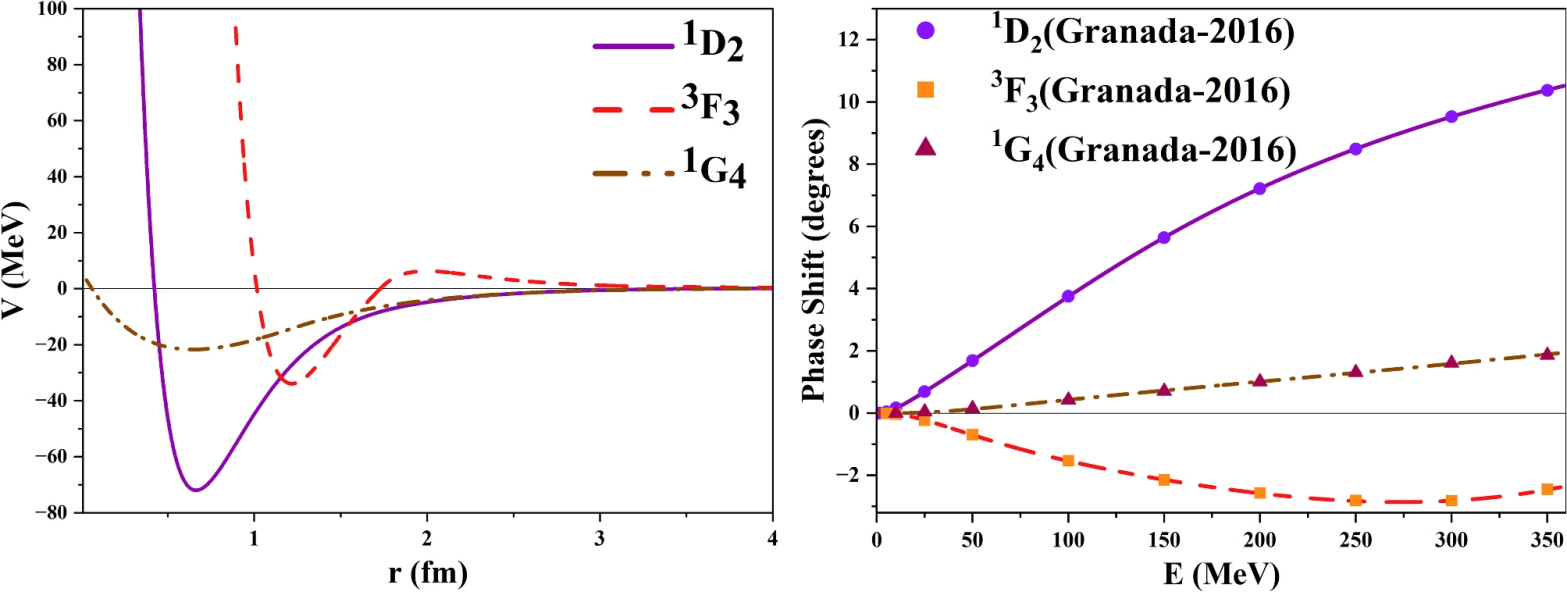
Inverse potentials and scattering phase shifts for the single channel scattering for D, F, and G wave after readjusting the bounds.

Hence, it is realized that the obtained inverse potentials for $^1D_2, ^3F_3,$ and $^1G_4$ states do not satisfy the physical considerations. Therefore, it was required to readjust the parameters to obtain more physically meaningful potentials. The new potentials for $^1D_2, ^3F_3,$ and $^1G_4$ states have converged to mean squared values of $10^{-4}$, 0.0003, 0.0001 respectively. These are smaller than those obtained previously and hence are leading to better global minima. The revised inverse potentials are plotted in Fig. 2 and show appropriate depths similar to those of neutron-proton states^21^. The final optimized potentials for all single-channel states are given in Table 1.

**Table 1:** Optimized parameters of inverse potentials for single channel proton–proton scattering states.

Parameters	$^1S_0$	$^3P_0$	$^3P_1$	$^1D_2$	$^3F_3$	$^1G_4$
$\alpha _0$	0.9352	0.2624	1.1717	2.9006	2.7703	0.8701
$\alpha _1$	3.2470	2.0049	1.2950	0.9182	2.6640	1.6697
$\alpha _2$	0.3873	0.5937	0.4714	0.4497	0.4382	0.4274
$r_0$	1.6330	1.8215	1.2894	0.6638	1.2184	0.6474
$r_1$	0.8545	0.0100	2.2307	11.0814	1.9960	0.4420
$r_2$	5.1215	18.0418	10.5225	19.9615	12.0030	19.5729
$X_1$	0.2207	1.9661	0.3249	1.8730	1.7436	1.2155
$X_2$	3.0429	3.3665	2.1280	4.1911	3.4865	4.4684
MSE $\times 10^{-3}$	1.7	0.2	0.8	$10^{-3}$	0.3	0.1

#### Multi-channel scattering

Typically mixing is observed for states with $\Delta L = 2$, having the same values of *J*. In the case of pp-scattering, we have only two possibilities with $J = 2$ and 4 that give rise to multi-channel scattering, those of ($^3P_2, ^3F_2$) and ($^3F_4, ^3H_4$). In these scenarios, the scattering phase shift data is given for individual *J*-states along with those for mixing parameters $\epsilon _J$. The phase shifts for multi-channel scattering are determined using equations for $\delta _{J, J-1}(r)$, $\delta _{J, J+1}(r)$ and $\epsilon _{J}(r)$ as given in Ref.^21^. Here, we optimize 30 parameters and obtain three inverse potentials corresponding to individual states and their mixing parameters.

#### Setting the bounds for solving coupled differential equations

**Fig. 3:**
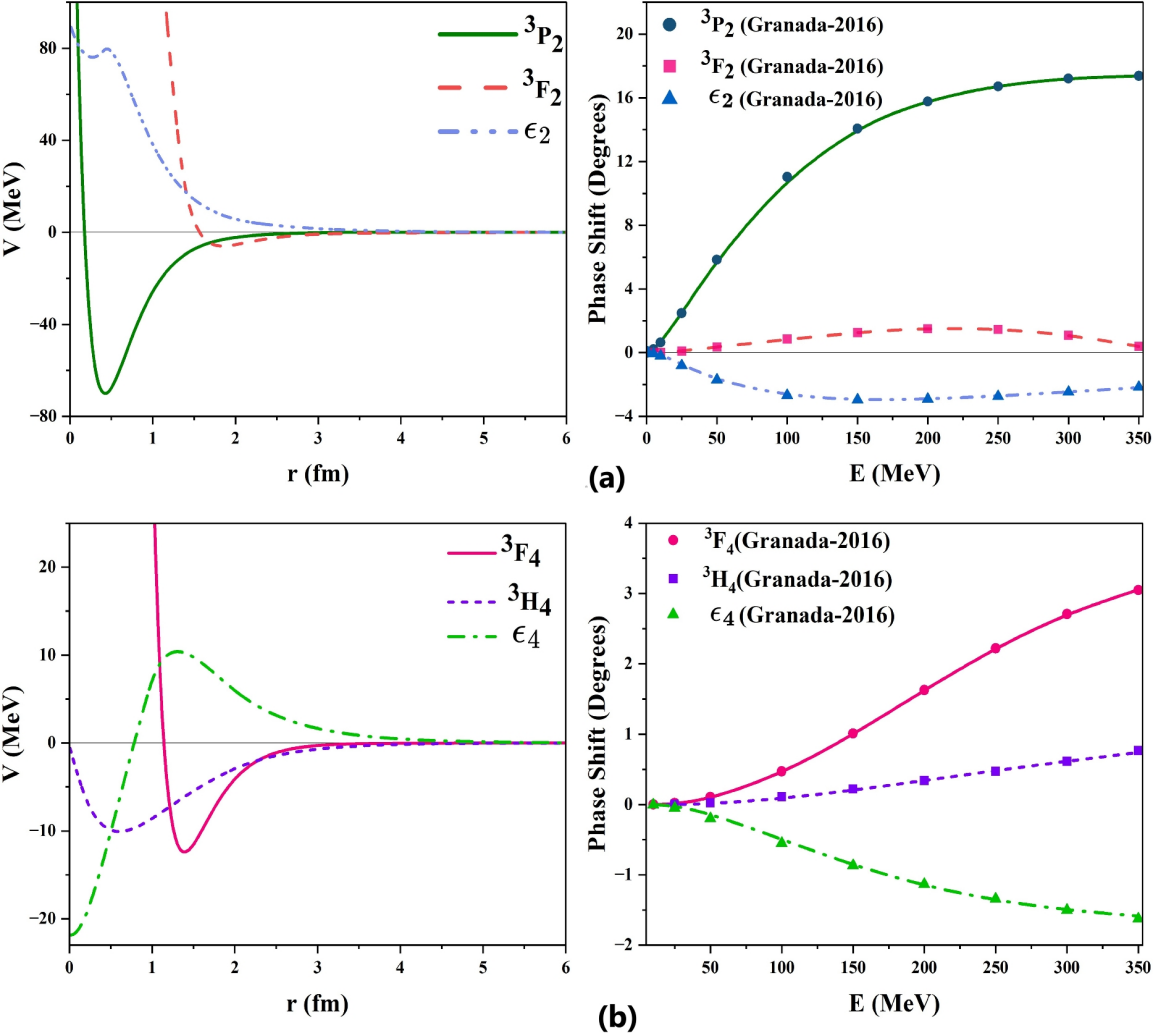
Inverse potentials and scattering phase shifts for the multi-channel scattering states with J = 2 (as in (**a**) and 4 (as in (**b**).

Solving three coupled equations for 30 parameters with large bounds, at 11 different energies simultaneously, would be an extremely time-consuming computational task. So, as a first step, the individual uncoupled phase equations were solved for each of the states ($^3P_2, ^3F_2, \epsilon _2$) and ($^3F_4, ^3H_4, \epsilon _4$) by choosing the initial bounds for various parameters from those of neutron-proton scattering study^21^. Now, we obtain three sets of 10 parameters for each mixing channel. The bounds for all 30 parameters can be defined as narrow intervals surrounding the values obtained from the independent channels. The three coupled equations are then solved simultaneously to determine the optimal set of 30 parameters that minimize the average of the three individual mean squared errors. The final optimized parameters, converged to mean squared error values to order $10^{-3}$, are given in Table 2. The constructed inverse potentials and corresponding phase shifts are shown in Fig. 2. The potentials for $^3P_2$ and $^3F_2$ exhibit attractive and repulsive parts as their phase shifts show an increasing trend till 300 *MeV* and then decrease. Phase shift values of mixing parameter $\epsilon _2$ are all negative, hence obtained tensor potential shows a repulsive nature. In the case of $^3F_4$, as shown in Right-top of Fig. 3, the phase shifts are positive with an increasing trend that tends to saturate, resulting in an inverse potential exhibiting an attractive character with a repulsion for short inter-nucleon distances. For the $^3H_4$ state, the phase shifts are positive over the entire range of energies and the resulting potential is attractive. The inverse potential for the mixing parameter $\epsilon _4$ interestingly shows a repulsive peak at almost the same distance where $^3F_4$ has a maximum depth of attraction.

**Table 2:** Optimized parameters for inverse potentials corresponding to multi-channel scattering states.

Parameters	$^3P_2$	$\epsilon _2$	$^3F_2$	$^3F_4$	$\epsilon _4$	$^3H_4$
$\alpha _0$	2.779	0.533	0.504	1.099	1.209	0.344
$\alpha _1$	0.960	2.353	2.504	2.794	1.390	1.126
$\alpha _2$	6.902	0.587	0.509	0.801	0.642	1.452
$r_0$	0.426	0.271	2.072	2.204	0.019	1.083
$r_1$	4.269	0.446	1.846	1.392	1.309	0.603
$r_2$	9.336	8.500	12.493	7.023	15.231	0.161
$X_1$	2.000	0.405	1.298	0.601	0.966	0.150
$X_2$	6.146	2.306	3.709	3.855	2.018	1.946

### Low energy scattering parameters

Low-energy parameters provide an effective framework for describing the dynamics of the scattering system. The computed low-energy scattering parameters, along with experimental data^35^ and results from the $Av_{18}$ potential^23^ and Slobodrian^36^, are presented in Table 3. The scattering length and effective range are calculated by including the electromagnetic interaction. In the realm of electromagnetic interaction, a more intricate effective range function (as per Eq. (4)) becomes necessary, wherein the phase shifts are about the entirety of the long-range electromagnetic interaction.

The *S*-wave effective-range expansion was used to examine scattering data for proton–proton interactions, including the Coulomb interaction. Slobodrian^36^ obtains the shape-dependent coefficients *P* and *Q* of the effective-range expansion based on empirical methods and ascertain their stability. The parameters *P* and *Q* have been calculated for various potential well shapes or models and are available in the literature^37^.

The scattering length and effective range values, for the A$v_{18}$ pp-potential, are given in Table 3 for comparison. It is important to mention that the A$v_{18}$ potential includes explicitly various Coulomb contributions, such as vacuum polarization, two-photon exchange, and magnetic moment interactions. Additionally, its short-range component is regulated through the utilization of dipole nucleon form factors. From Table 3, we can observe that results obtained using our reference potential approach match the A$v_{18}$ values, remarkably.

**Table 3:** Low-energy scattering parameters for proton-proton scattering obtained using this work based on reference potential approach in comparison with those obtained from A$v_{18}$ potential and experimental ones.

	Experiment^35^	A$v_{18}$^23^	Slobodrian^36^	Our work
$a_{p}(fm)$	$-7.8063 \pm 0.0026$	$-7.8064$	$-7.7856$	$-7.7699$
$r_{e}(fm)$	$2.7940 \pm 0.0140$	2.7880	2.8400	2.7807
*P*	–	–	0.0720	0.0460
*Q*	–	–	0.0340	0.0390

### Scattering cross-sections

Finally, we have computed the partial and total scattering cross-sections using the obtained phase shifts.

*Partial cross-section* For all the states *S*, *P*, *D*, *F*, *G*, and *H* partial cross-sections have been calculated using Eq. (8) at various lab energies and are presented in Table 4. The $\%$-contribution due to the individual states, of all $\ell$-channels from *S* to *H*, to the calculated total scattering cross-section are given in brackets. One can observe that the $^1S_0$ state has a large contribution at low energies and then it gradually falls with increasing energy and becomes very less beyond 100 *MeV*. The contributions from *P* and *D* channels become significant for higher energies ranging from 100 to 350 *MeV*. Those due to *F* and *G* states are far less in comparison within the same range but certainly become more important for accurately describing the observed experimental total scattering cross-section. The scattering phase shift for the *H*-state being very small, its contribution to the total cross section is almost negligible.

**Table 4:** The individual contributions to the calculated total elastic scattering cross-section (SCS) from various $\ell$ channels. Their $\%$-contributions have been given in brackets.

*E* (*MeV*)	$\sigma _S$	$\sigma _P$	$\sigma _D$	$\sigma _F$	$\sigma _G$	$\sigma _H$	$\sigma _{total}$ (barn)	$\sigma _{exp}$ (barn)
1	1.520 ($100\%$)	$2.67\times 10^{-5}$ ($0\%$)	$4.77\times 10^{-9}$	0	0	0	1.520	1.513
5	0.698 ($99.92\%$)	0.0005 ($0.08\%$)	$1.60\times 10^{-6}$	$1.34\times 10^{-8}$	0	0	0.698	0.691
10	0.353 ($99.57\%$)	0.0015 ($0.42\%$)	$1.20\times 10^{-5}$	$3.16\times 10^{-7}$	$1.43\times 10^{-9}$	0	0.355	0.350
25	0.118 ($96.9\%$)	0.0037 ($3.02\%$)	$9.08\times 10^{-5}$ ($0.07\%$)	$8.79\times 10^{-6}$ ($0.01\%$)	$3.57\times 10^{-7}$	0	0.122	0.127
50	0.042 ($88.21\%$)	0.0052 ($11.11\%$)	0.0003 ($0.57\%$)	$4.52\times 10^{-5}$ ($0.1\%$)	$4.83\times 10^{-6}$ ($0.01\%$)	$4.13\times 10^{-8}$	0.047	0.059
100	0.010 ($57.4\%$)	0.0063 ($37.74\%$)	0.0007 ($3.99\%$)	0.0001 ($0.71\%$)	$2.64\times 10^{-5}$ ($0.16\%$)	$1.18\times 10^{-6}$ ($0.01\%$)	0.017	0.032
150	0.002 ($23.34\%$)	0.0066 ($64.54\%$)	0.0010 ($9.85\%$)	0.0001 ($1.73\%$)	$5.04\times 10^{-5}$ ($0.49\%$)	$4.77\times 10^{-6}$ ($0.05\%$)	0.010	0.026
200	0.0004 ($4.54\%$)	0.0066 ($77.49\%$)	0.0012 ($14.38\%$)	0.0002 ($2.62\%$)	$7.29\times 10^{-5}$ ($0.85\%$)	$1.02\times 10^{-5}$ ($0.12\%$)	0.009	0.024
250	$2.57\times 10^{-7}$	0.0066 ($79.27\%$)	0.0014 ($16.39\%$)	0.0002 ($3\%$)	$9.54\times 10^{-5}$ ($1.15\%$)	$1.62\times 10^{-5}$ ($0.19\%$)	0.008	0.024
300	0.0002 ($2\%$)	0.0065 ($76.57\%$)	0.0014 ($16.87\%$)	0.0002 ($2.88\%$)	0.0001 ($1.41\%$)	$2.21\times 10^{-5}$ ($0.26\%$)	0.009	0.024
350	0.0005 ($6.06\%$)	0.0063 ($72.79\%$)	0.0015 ($16.73\%$)	0.0002 ($2.44\%$)	0.0002 ($1.67\%$)	$2.74\times 10^{-5}$ ($0.32\%$)	0.009	0.025

#### Total scattering cross section

We have calculated the total scattering cross-sections using Eq. (9) by summing over the contribution of all the states of *S*, *P*, *D*, *F*, *G*, and *H*.

**Fig. 4:**
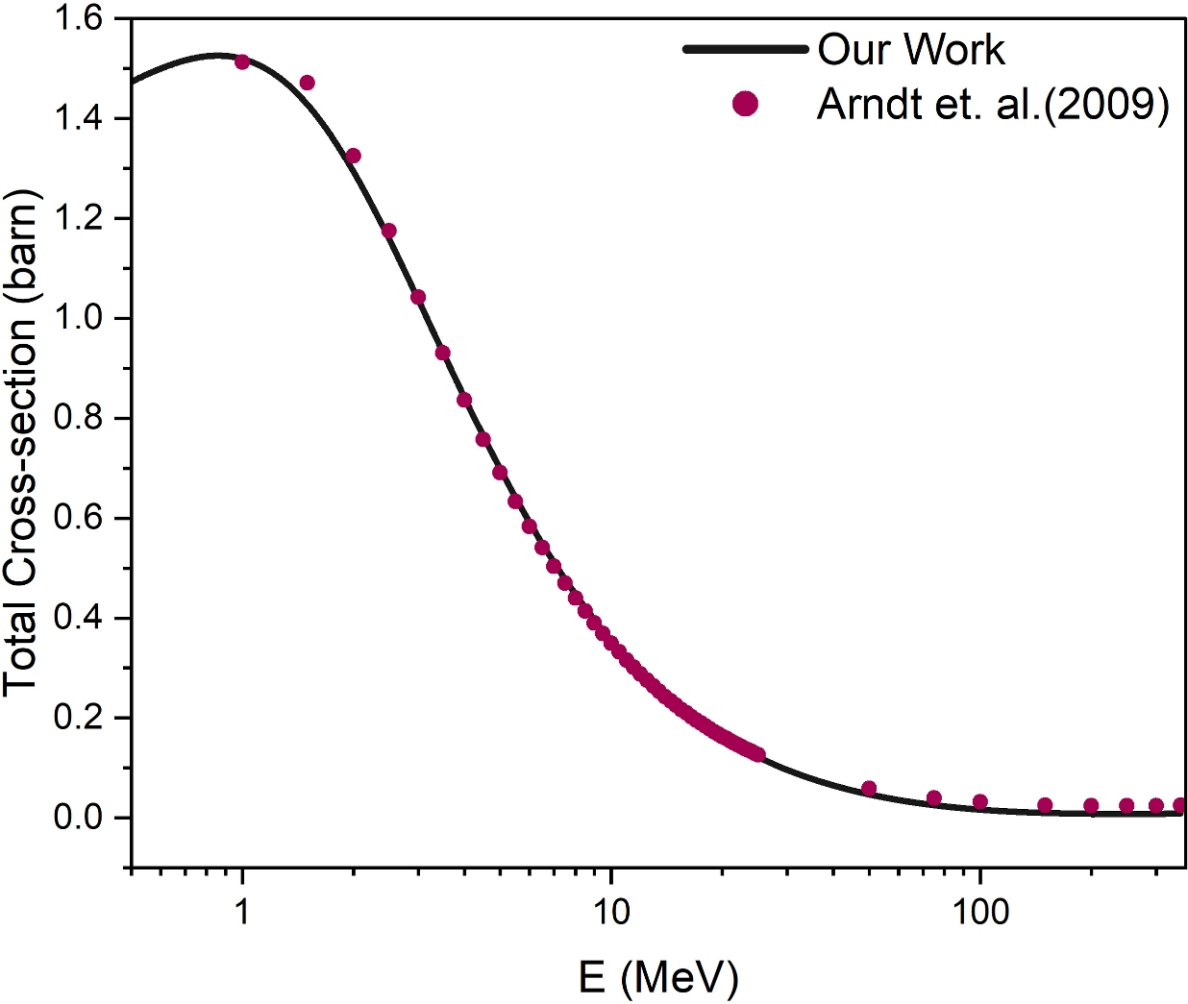
Total scattering cross-section along with the experimental data^38^ plotted on a log scale.

The total SCS has been plotted along with experimental data of Arndt et al.^38^ for the lab energies, up to 350 *MeV*, on a log scale in Fig. 4. Overall the obtained total scattering cross-sections are found to be closely matched with the experimental ones. One can observe that the $^1S_0$ state contributes significantly at energies below 5 *MeV* and decreases progressively as energy increases.

## Conclusions

We have fully resolved the issue of incorporating Coulomb interaction in charged particle systems phenomenologically without resorting to any approximations. The resulting inverse potentials are found to be very similar to those of our neutron-proton ones. The exact interaction potentials corresponding to various J-states of proton-proton scattering are explicitly plotted for the first time. A key factor in successfully constructing the inverse potential is the formulation of the reference function. The combination of smoothly joined regular and reversed Morse functions across different regions of inter-nuclear distances effectively describes the nature of nuclear and Coulomb interactions in charged particle systems.

This research shows that the phase function approach is very effective in constructing inverse potential for fixed-$\ell$ methods. The main advantage of this approach is that one can directly obtain the scattering phase shifts, deduced from the experimental scattering cross-sections, by solving the phase equations for a chosen input potential. Since the phase equation is a non-linear first-order differential equation with the potential appearing only as a multiplicative function, it is important to consider a class of smooth or piece-wise smooth functions for constructing the reference input. The final factor that contributes to the success of the procedure is the optimization routines, which are part of various libraries, available in free open-source software such as Python and Scilab. In conclusion, our novel approach for constructing inverse potential for charge particle systems using piece-wise smooth reference potential is an efficient way to incorporate screened Coulomb potential into the model. The scattering parameters and cross-sections obtained for pp-scattering closely align with the experimental ones, thus validating our approach.

## Data Availability

The datasets used and/or analyzed during the current study are available from the Granada Database. https://www.ugr.es/~amaro/nndatabase/database.php
